# Clinical phenotype identification based on inflammation–nutrition–coagulation biomarkers in advanced non-small cell lung cancer

**DOI:** 10.3389/fnut.2026.1839434

**Published:** 2026-07-15

**Authors:** Huanhuan Zhao, Yan Wang, Shaoyang Zhao

**Affiliations:** 1 Department of Nutrition, Linyi People’s Hospital, The Affiliated Hospital of Shandong Second Medical University, Linyi, Shandong, China; 2 Department of Medical Record Management, Linyi People’s Hospital, The Affiliated Hospital of Shandong Second Medical University, Linyi, Shandong, China; 3 Department of Endocrinology, Linyi People’s Hospital, The Affiliated Hospital of Shandong Second Medical University, Linyi, Shandong, China

**Keywords:** inflammation–nutrition–coagulation axis, K-means clustering, non-small cell lung cancer, phenotype stratification, principal component analysis

## Abstract

**Objective:**

This study aimed to identify clinically distinct host phenotype subgroups based on inflammation–nutrition–coagulation biomarkers in patients with advanced non-small cell lung cancer (NSCLC). It also evaluated their association with conventional Tumor–Node–Metastasis (TNM) staging.

**Methods:**

A total of 1,644 patients with advanced NSCLC were retrospectively included. Twenty-two baseline laboratory indicators spanning inflammation, nutrition, and coagulation were collected. After data cleaning and Z-score standardization, principal component analysis (PCA) was performed for dimensionality reduction. The optimal number of clusters was determined using the elbow method, silhouette coefficient, and Calinski–Harabasz index.

**Results:**

The Kaiser–Meyer–Olkin value was 0.631, and Bartlett’s test of sphericity was significant (*p* < 0.001), confirming data suitability for PCA. Nine principal components were extracted, explaining 80.18% of the cumulative variance. K-means clustering identified three phenotype subgroups. Subgroup 1 (*n* = 113, 6.87%) showed high-risk pattern characterized by severe inflammation, poor nutritional status, and hypercoagulability. Subgroup 3 (*n* = 521, 31.69%) represented a favorable phenotype with low inflammation, adequate nutrition, and low coagulation activity. Subgroup 2 (*n* = 1,010, 61.44%) showed intermediate characteristics. This classification was not associated with TNM staging (*p* = 0.578).

**Conclusion:**

PCA combined with K-means clustering identified three distinct host phenotype subgroups along the inflammation–nutrition–coagulation axis in advanced NSCLC. These subgroups were independent of TNM staging and provide a new framework for individualized risk stratification and clinical management.

## 1 Introduction

Lung cancer is one of the leading causes of cancer-related mortality worldwide, among which non-small cell lung cancer (NSCLC) accounts for approximately 85% of all lung cancer cases (1). Despite standardized treatment, patients with advanced NSCLC exhibit significant heterogeneity in clinical outcomes. This heterogeneity stems from complex interactions between tumor biology and host physiology, and represents a core challenge in precision oncology (2).

Malnutrition is one of the most common and clinically significant complications in patients with advanced NSCLC. Studies have shown that the prevalence of malnutrition and nutritional risk is high among patients with advanced lung cancer and is closely associated with reduced treatment tolerance, increased complications, prolonged hospital stay, decreased quality of life, and poor prognosis (3–5). Therefore, early identification of nutritional risk and implementation of individualized nutritional interventions have become important components of comprehensive cancer management. However, the nutritional status of patients with cancer is not determined solely by nutritional intake, but is jointly influenced by multiple factors, including inflammatory responses, metabolic abnormalities, and host–tumor interactions; traditional nutritional assessment methods often fail to comprehensively reflect patients’ actual physiological status.

Host phenotype integrates multidimensional indicators, including physiological, biochemical, and immunological parameters, and reflects the systemic functional status of the host within the tumor microenvironment (6). Within this framework, inflammation, nutrition, and coagulation constitute the three core response systems of the host to tumors. Dysregulation of these systems is involved in tumor progression and directly affects treatment tolerance (7). Notably, cancer-related malnutrition is not simply insufficient nutrient intake, but a metabolic disorder syndrome centered on systemic inflammation, characterized by enhanced protein catabolism, depletion of visceral proteins, impaired immune function, and coagulation abnormalities (8). An increasing number of studies have shown that inflammatory markers (NLR, CRP), nutritional markers (albumin, PNI), and coagulation markers (such as D-dimer) are closely interrelated and jointly reflect patients’ nutritional reserves, metabolic status, and disease burden (9, 10). Therefore, a comprehensive assessment based on the inflammation–nutrition–coagulation axis may enable a more comprehensive identification of nutritional risk and host status in patients with advanced NSCLC than a single indicator.

Previous studies have largely focused on associations between individual or limited indicators and clinical outcomes, and have not captured the interactions among the inflammation, nutrition, and coagulation systems. To date, no study has integrated the above multi-dimensional indicators and used unsupervised clustering algorithms to explore host phenotype subgroups in patients with advanced NSCLC. As an exploratory analytical method for high-dimensional data, unsupervised clustering can effectively mine the potential intrinsic structure of data (11, 12). In this study, principal component analysis (PCA) and K-means clustering were used solely as analytical tools to identify patient subgroups with different host states and nutritional risk profiles, with the aim of extracting clinically meaningful latent phenotypes from multidimensional indicators.

## 2 Research subjects and methods

### 2.1 Research subjects

This retrospective cohort study enrolled patients with advanced NSCLC diagnosed at Linyi People’s Hospital between January 2023 and December 2025. All cases were confirmed by histopathology or cytology. Disease stage was determined according to the AJCC 8th edition criteria.

The inclusion criteria were as follows: (1) confirmed diagnosis of NSCLC; (2) stage III–IV disease; (3) complete baseline clinical and laboratory data; (4) no history of other malignancies; and (5) no active infection, autoimmune disease, or severe organ dysfunction (defined as ALT/AST > 3 × the upper limit of normal for liver, or serum creatinine >2 × the upper limit of normal for kidney). Exclusion criteria were: (1) had received any anti-tumor treatment before admission; (2) comorbidities potentially confounding inflammatory, nutritional, or coagulation parameters (e.g., hematological disorders, severe cardiovascular disease); (3) > 20% missing data for required variables; or (4) extreme laboratory outliers (|Z| > 3). This study was approved by the Ethics Committee of Linyi People’s Hospital (Approval No. 202603-H-001). All procedures were conducted in accordance with the Declaration of Helsinki. As this was a retrospective study using anonymized clinical data, the requirement for informed consent was waived.

### 2.2 Data collection

Clinical and laboratory data were retrospectively obtained from the hospital’s electronic medical record system. All laboratory tests were performed within 1 week after admission and prior to the initiation of anti-tumor treatment. In addition, standardized nutritional screening information at admission was extracted from clinical nutrition-related records. Nutritional Risk Screening 2002 (NRS-2002) was used to assess nutritional risk at admission, and an NRS-2002 score of ≥3 was defined as the presence of nutritional risk. As this was a retrospective study, information related to the PG-SGA, MUST, and GLIM criteria was not completely recorded for all patients and was therefore not included in the primary analysis. The 22 baseline variables were selected based on established clinical evidence and biological plausibility within the inflammation–nutrition–coagulation framework. Inflammatory indicators (NLR, platelet-to-lymphocyte ratio [PLR], systemic immune-inflammation index [SII], C-reactive protein [CRP]) were included for their well-documented independent prognostic value in NSCLC (13, 14). Nutritional parameters (albumin, total protein, PNI, body mass index [BMI], albumin-to-globulin ratio [A/G]) reflect the metabolic reserve and immune function of the host (8, 15). Coagulation markers (fibrinogen, D-dimer) capture the hypercoagulable state associated with malignancy (16). Hematological parameters (complete blood counts) and tumor markers (carcinoembryonic antigen [CEA], neuron-specific enolase [NSE], cytokeratin 19 fragment [CYFRA 21-1]) were included to characterize the host–tumor interaction.

The 22 variables were organized into four categories: (1) demographic and clinical characteristics (age, sex, smoking status, alcohol consumption, histological subtype, clinical stage, BMI, cachexia status); (2) hematological and biochemical indicators (white blood cell count [WBC], red blood cell count [RBC], hemoglobin, platelet count, neutrophil count, lymphocyte count, CRP, total protein, albumin, globulin, fibrinogen, D-dimer, CEA, NSE, CYFRA 21-1); (3) systemic inflammation indices (NLR, PLR, SII, fibrinogen-to-albumin ratio index [FARI]); (4) nutritional prognostic indicators (A/G, PNI). The PNI was calculated as follows: albumin (g/L) + 5 × lymphocyte count (×10^9^/L).

Cachexia was defined according to the international consensus criteria for cancer cachexia (2), namely, involuntary weight loss of >5% within the previous 6 months; or a BMI of <20 kg/m^2^ and involuntary weight loss of >2% within the previous 6 months; or the presence of reduced muscle mass accompanied by weight loss of >2%. As this was a retrospective study, muscle mass data were not available for all patients; therefore, the operational determination was based primarily on weight changes and BMI recorded in the electronic medical records.

### 2.3 Data preprocessing

A standardized preprocessing procedure was applied before statistical analysis to improve data quality and reliability. Missing values in continuous variables were imputed using the median, and missing categorical variables were imputed using the mode. All retained variables had missing rates below 20%. The only variable with relatively notable missingness was sex (11.2%), which was a categorical variable not included in the PCA procedure. Missing values among continuous variables were minimal and remained below the predefined threshold; therefore, their influence on estimation of the sample covariance matrix and subsequent PCA-based dimensionality reduction was expected to be negligible. Although multiple imputation better preserves data variability under missing-at-random assumptions (17, 18), its additional complexity was considered unnecessary for this exploratory analysis. Outliers in continuous variables were identified using the Z-score method, with a threshold of |Z| > 3 (16, 17). When outlier removal exceeded 10% of observations for a given variable, Winsorization was applied to limit the influence of extreme values by replacing extreme values with ±3*σ* bounds. All continuous variables were standardized using Z-score normalization (Z = (X − *μ*) / σ). Categorical variables were excluded from PCA and clustering but retained for descriptive subgroup comparisons.

### 2.4 Principal component analysis

PCA was performed to reduce the dimensionality of the dataset and to decrease multicollinearity among variables. First, the suitability of the data for PCA was evaluated using the Kaiser–Meyer–Olkin (KMO) test and Bartlett’s test of sphericity. A KMO value above 0.6 was considered acceptable, based on the classification proposed by Kaiser (19). A significant Bartlett’s test of sphericity (*p* < 0.05) confirmed sufficient inter-variable correlations for PCA (20). Second, principal components were extracted based on eigenvalues greater than 1 and a cumulative explained variance of at least 80% (21). Third, the component loading matrix was analyzed. Loadings with an absolute value ≥ 0.4 were considered strong associations, and loadings between 0.3 and 0.4 were considered moderate associations (22). These results were combined with biological meaning to interpret the principal components.

### 2.5 K-means clustering analysis

K-means clustering was performed using the principal component scores as input. The optimal number of clusters (k) was determined using three complementary metrics: the elbow method (identifying the inflection point of the inertia curve), the silhouette coefficient (higher values closer to 1 indicate better cluster quality), and the Calinski–Harabasz (CH) index (higher values preferred). Patients were assigned to subgroups based on the optimal k. Clustering validity was confirmed by an average silhouette coefficient above 0.

### 2.6 Statistical analysis

Continuous variables with a normal distribution were expressed as mean ± standard deviation (SD). Group comparisons were performed using one-way analysis of variance (ANOVA). Variables with a non-normal distribution were presented as median with interquartile range [M (Q1, Q3)]. Differences among groups were assessed using the Kruskal–Wallis H test. Categorical variables were summarized as counts and percentages [n (%)]. Comparisons between groups were performed using the chi-square test. Pearson correlation analysis was used to examine relationships between variables. A correlation heatmap was generated to visualize these associations. Sensitivity analyses were conducted for missing sex data: the χ^2^ test was used to compare differences in the rate of missing sex data among the different phenotypic subgroups, and differences in sex distribution among the subgroups were re-examined among patients with known sex. A two-sided significance level was set at *α* = 0.05. A *p* value < 0.05 was considered statistically significant. All analyses and visualizations were performed using Python (version 3.9). Matplotlib and Seaborn were applied for graphical presentation. Statistical tests were conducted using SciPy and Scikit-learn.

## 3 Results

### 3.1 Data preprocessing results

A total of 2019 patients with advanced NSCLC were initially enrolled. After missing data imputation and outlier filtering, 1,644 patients were included in the final analysis, corresponding to a retention rate of 81.4%. The detailed data cleaning procedure is summarized in Table 1.

**Table 1:** Data processing and sample retention.

Processing stage	Sample size (n)	Retention rate (%)
Original included data	2019	100.0
After missing value imputation	2019	100.0
After outlier filtering	1,644	81.4

Outlier filtering was performed using the Z-score method (|Z| > 3). For variables where outliers exceeded 10% of the total sample, Winsorization was applied by replacing extreme values with ±3σ boundary values; otherwise, outlier records were directly excluded.

### 3.2 Baseline characteristics of the study population

Baseline demographic, clinical, and laboratory characteristics are summarized in Table 2. Of the 1,644 patients, 875 (53.2%) were male and 585 (35.6%) were female, while sex information was missing for 184 patients (11.2%) owing to incomplete documentation in the electronic medical record system. Regarding histopathological subtype, squamous cell carcinoma was the most common type (1,058 cases, 64.4%), followed by adenocarcinoma (352 cases, 21.4%) and other subtypes (234 cases, 14.2%). The majority of patients were diagnosed at clinical stage III (647 cases, 39.4%) or stage IV (975 cases, 59.3%). The mean age of the study population was 66.84 ± 9.89 years, and the prevalence of cancer cachexia was 25.1% (413/1644).

**Table 2:** Baseline characteristics of the study population (*n* = 1,644).

Indicator type	Variable	Statistical value
Continuous variables, mean ± SD	Age (years)	66.84 ± 9.89
	White blood cell count (×10^9^/L)	8.14 ± 3.63
	Red blood cell count (×10^12^/L)	4.33 ± 0.56
	Neutrophil count (×10^9^/L)	5.86 ± 3.20
	Hemoglobin (g/L)	129.35 ± 16.96
	Platelet count (×10^9^/L)	260.49 ± 85.59
	Lymphocyte count (×10^9^/L)	1.52 ± 0.65
	Total protein (g/L)	67.29 ± 6.63
	Albumin (g/L)	39.56 ± 4.78
	Globulin (g/L)	27.67 ± 4.93
	Fibrinogen (g/L)	4.54 ± 1.47
	D-dimer (ng/mL)	142.08 ± 283.88
	CEA (ng/mL)	24.96 ± 14.26
	NSE (ng/mL)	33.04 ± 41.77
	CYFRA 21-1 (ng/mL)	36.04 ± 70.68
	CRP (mg/L)	91.92 ± 100.59
	BMI (kg/m^2^)	22.85 ± 7.09
	NLR	4.90 ± 4.95
	PLR	203.92 ± 119.87
	SII	1275.47 ± 1310.51
	FARI	0.11 ± 0.05
	A/G	1.47 ± 0.31
	PNI	47.09 ± 6.32
Categorical variables, n (%)	Gender	**–**
	Male	875 (53.2)
	Female	585 (35.6)
	Unknown	184 (11.2)
	Pathological type	**–**
	Squamous cell carcinoma	1,058 (64.4)
	Adenocarcinoma	352 (21.4)
	Other	234 (14.2)
	Tumor stage	**–**
	Stage II	22 (1.3)
	Stage III	647 (39.4)
	Stage IV	975 (59.3)
	Smoking history	**–**
	Yes	781 (47.5)
	No	863 (52.5)
	Drinking history	**–**
	Yes	572 (34.8)
	No	1,072 (65.2)
	Cachexia	**–**
	Yes	413 (25.1)
	No	1,231 (74.9)

NLR, PLR, SII, FARI, A/G, and PNI are dimensionless ratio indices. NLR, Neutrophil-to-lymphocyte ratio; PLR, Platelet-to-lymphocyte ratio; SII, Systemic immune-inflammation index; FARI, Fibrinogen-to-albumin ratio index; A/G, Albumin-to-globulin ratio; PNI, Prognostic nutritional index; CEA, Carcinoembryonic antigen; NSE, Neuron-specific enolase; CYFRA 21-1, Cytokeratin 19 fragment; CRP, C-reactive protein. “Other” types included NSCLC-not otherwise specified (NSCLC-NOS) and other rare histological subtypes of non-small cell lung cancer not classified as adenocarcinoma or squamous cell carcinoma. Due to the limited number of cases, these patients were not analyzed as an independent subgroup.

### 3.3 Correlation analysis of core indicators

**Figure 1:**
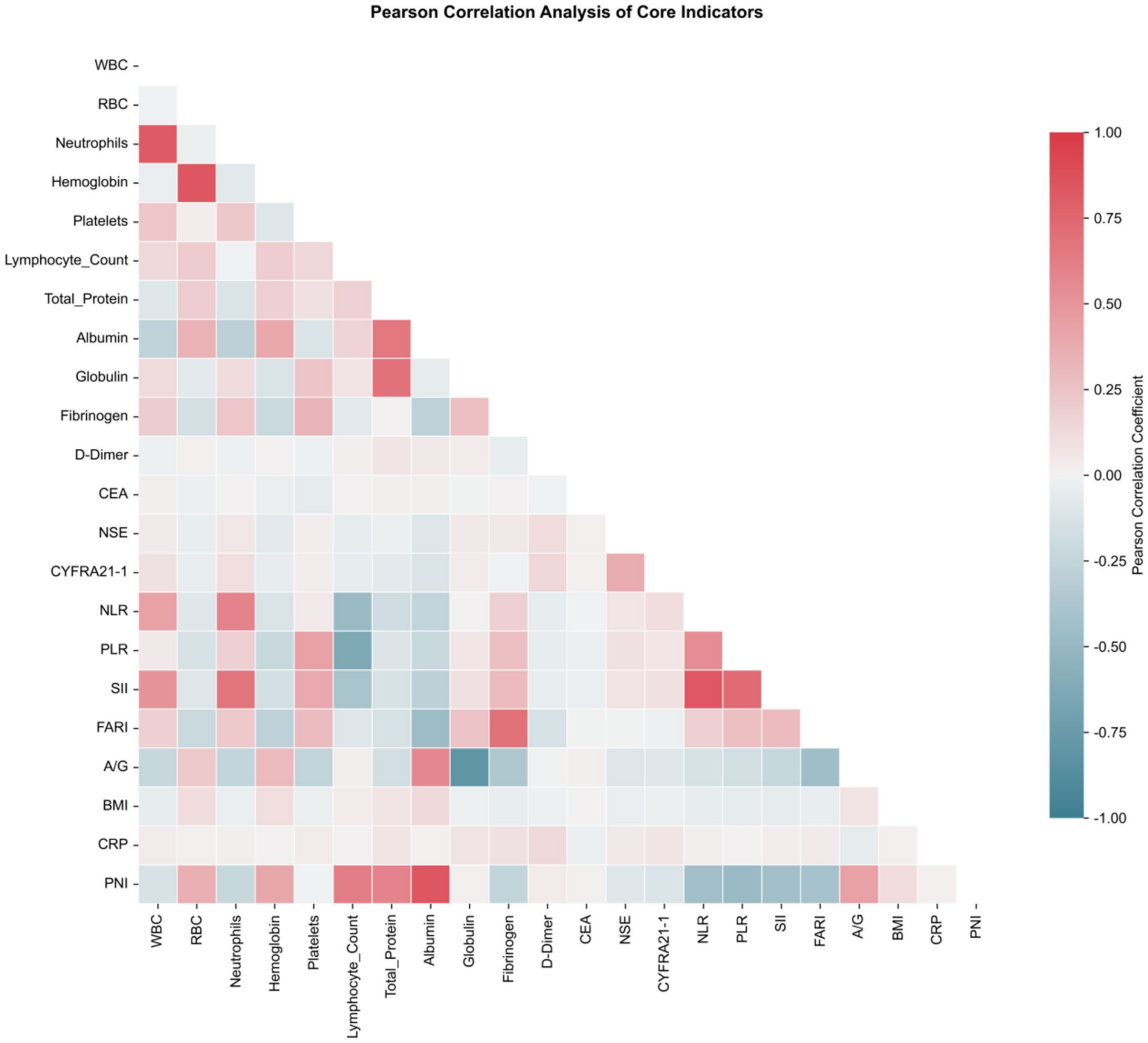
Pearson correlation analysis of core indicators in advanced NSCLC. Red denotes positive correlations and blue denotes negative correlations, with color intensity proportional to the absolute correlation coefficient. The diagonal elements represent self-correlations (coefficient = 1). NLR, neutrophil-to-lymphocyte ratio; PLR, platelet-to-lymphocyte ratio; SII, systemic immune-inflammation index; FARI, fibrinogen-to-albumin ratio index; A/G, albumin-to-globulin ratio; PNI, prognostic nutritional index; CRP, C-reactive protein; CEA, carcinoembryonic antigen; NSE, neuron-specific enolase; CYFRA 21-1, cytokeratin 19 fragment; WBC, white blood cell count; RBC, red blood cell count.

Pearson correlation analysis revealed significant multicollinearity among variables within the three systems (r > 0.70), supporting the use of PCA for dimensionality reduction (Figure 1). Strong positive correlations were found among inflammatory indicators (NLR, PLR, SII) and among nutritional indicators (albumin, PNI, total protein). Fibrinogen and D-dimer showed weak to moderate correlations with inflammatory indicators, suggesting crosstalk between the inflammation and coagulation systems and supporting the rationale for integrated analysis. Tumor markers showed weak correlations with both inflammatory and nutritional indicators (|r| < 0.25), indicating that they carry information independent of host status.

### 3.4 Results of principal component analysis

#### 3.4.1 PCA adequacy testing

The KMO value was 0.631, and Bartlett’s test of sphericity was significant (χ^2^ = 30,965.06, *p* < 0.001), confirming that the data were suitable for PCA (Supplementary Table 1).

#### 3.4.2 Extraction of principal components

Based on the Kaiser criterion (eigenvalues > 1) and a cumulative explained variance threshold of ≥ 80%, nine principal components were retained. These components collectively accounted for 80.18% of the total variance (Supplementary Table 2).

**Figure 2:**
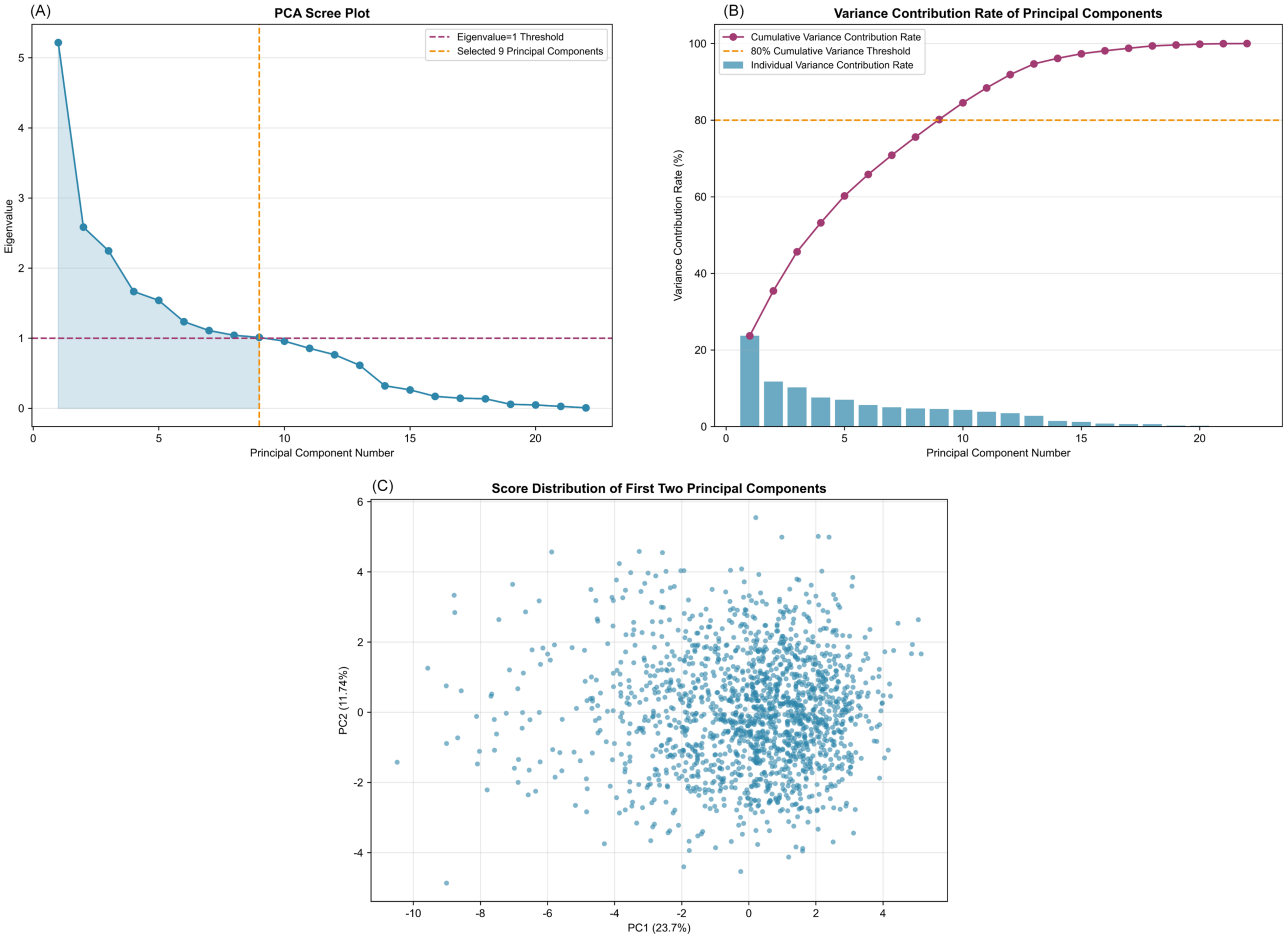
PCA dimensionality reduction diagnostics and score distribution of the first two principal components. **(A)** Scree plot of eigenvalues. The blue line shows the trend of eigenvalues for each principal component; the purple dashed line indicates the eigenvalue = 1 threshold based on the Kaiser criterion; the orange dashed line marks the nine retained components; and the light blue shaded area highlights the variance explanation range of the first nine components; **(B)** Individual and cumulative variance explained by principal components. Blue bars denote the individual explained variance of each component; the purple line indicates the cumulative explained variance; and the orange dashed line marks the 80% threshold; **(C)** Score plot of patients on the PC1–PC2 plane. Each point represents one patient, and the x- and y-axes show PC1 and PC2 scores, respectively.

The scree plot (Figure 2A) illustrated a sharp decline in eigenvalues for the first three components (ranging from 5.217 to 2.247). These components represented the most dominant dimensions of the dataset. Beyond the third component, the curve gradually flattened and intersected the eigenvalue = 1 reference line at the ninth component, satisfying the extraction criteria. Furthermore, the variance contribution analysis (Figure 2B) confirmed that the first principal component (PC1) explained 23.70% of the total variance, serving as the primary source of phenotypic variation. The cumulative variance curve exhibited a distinct inflection point at the ninth component, where it surpassed the 80% threshold, thereby validating the rationale for retaining nine principal components.

#### 3.4.3 Biological interpretation of principal components

The loading matrix (Table 3) revealed four interpretable physiological modules. The inflammation module was driven by PC1, PC4, and PC8. PC1 had core loadings on SII (−0.341) and PLR (−0.285), and PC8 had a primary loading on CRP (0.609), reflecting the interaction between systemic immune activation and the acute-phase response. The nutrition module centered on PC2, with primary loadings on globulin (0.499) and total protein (0.471); PC9 was represented by BMI (0.555), capturing protein metabolism homeostasis and nutritional reserve. The coagulation module was led by PC3. PC5 had a core loading on D-dimer (0.416), PC6 was represented by red blood cell count (0.455) and hemoglobin (0.403), and PC7 had a primary loading on platelets (0.493). In the tumor marker module, PC5 was characterized by NSE (0.531) and CYFRA 21-1 (0.567), and PC9 was represented by CEA (0.619). FARI showed low loadings across all components and served as a minor supplementary indicator.

**Table 3:** Loading matrix of the first 9 principal components.

Indicator	PC1	PC2	PC3	PC4	PC5	PC6	PC7	PC8	PC9
White blood cell count	−0.209	0.187	0.304	0.409*	0.041	−0.209	0.009	0.054	0.016
Red blood cell count	0.184	0.150	0.372	0.002	−0.073	0.455	−0.290	−0.05	−0.034
Neutrophil count	−0.258	0.150	0.346	0.301	0.043	−0.219	−0.030	0.059	0.032
Hemoglobin	0.220	0.104	0.370	0.011	−0.064	0.403*	−0.313	−0.017	−0.025
Platelet count	−0.155	0.284	0.063	−0.049	−0.148	0.207	0.493*	−0.174	−0.187
Lymphocyte count	0.200	0.282	−0.052	0.488*	−0.032	0.019	0.214	−0.056	−0.066
Total protein	0.153	0.471*	−0.011	−0.344	0.079	−0.218	−0.067	−0.01	0.003
Albumin	0.318	0.144	0.211	−0.305	0.001	−0.193	0.210	−0.035	0.067
Globulin	−0.100	0.499*	−0.214	−0.172	0.104	−0.117	−0.300	0.017	−0.073
Fibrinogen	−0.233	0.215	−0.125	0.001	−0.204	0.256	0.204	−0.032	0.229
D-dimer	0.030	0.030	−0.006	−0.028	0.416*	0.049	0.151	0.369	0.041
CEA	0.008	−0.006	−0.022	0.034	0.037	−0.215	−0.075	−0.498*	0.619*
NSE	−0.066	0.004	−0.009	0.002	0.531*	0.225	0.103	−0.341	−0.011
CYFRA 21-1	−0.072	−0.012	0.032	0.050	0.567*	0.195	0.058	−0.245	−0.006
NLR	−0.296	−0.068	0.350	−0.129	0.044	−0.178	−0.127	0.069	0.055
PLR	−0.285	−0.057	0.143	−0.458*	−0.065	0.107	0.165	−0.102	−0.078
SII	−0.341	0.041	0.345	−0.139	−0.015	−0.096	0.087	−0.002	−0.03
FARI	−0.267	0.138	−0.191	0.066	−0.265	0.277	0.069	−0.042	0.182
A/G	0.252	−0.322	0.279	−0.033	−0.088	−0.024	0.362	−0.033	0.100
BMI	0.063	0.035	0.066	−0.052	−0.069	0.098	−0.097	0.040	0.555*
CRP	−0.016	0.093	−0.004	−0.042	0.194	0.184	0.189	0.609*	0.387
PNI	0.345	0.261	0.133	0.013	−0.021	−0.148	0.272	−0.062	0.009
Explained variance (%)	23.7	11.7	10.2	7.6	7.0	5.6	5.0	4.7	4.6

Absolute loadings ≥ 0.40 are considered strong associations and are marked with an asterisk (*); loadings between 0.3 and 0.4 are considered moderate associations; FARI, fibrinogen-to-albumin ratio index; A/G, albumin-to-globulin ratio.

#### 3.4.4 Distribution of principal component scores

The PCA score plot for the first two principal components (PC1 and PC2), accounting for 35.44% of the total variance, revealed a predominantly homogeneous distribution of patient phenotypes (Figure 2C). The majority of samples were densely clustered around the origin, indicative of a core phenotypic pattern. In contrast, a few outlying samples were dispersed away from the main cluster, suggesting the existence of extreme phenotypic subgroups. Notably, the data spread along PC1 (explaining 23.70% of the variance) was substantially greater than that along PC2 (11.74%), confirming PC1 as the primary dimension driving phenotypic heterogeneity in this cohort.

### 3.5 Results of K-means clustering analysis

#### 3.5.1 Determination of the optimal number of clusters

**Figure 3:**
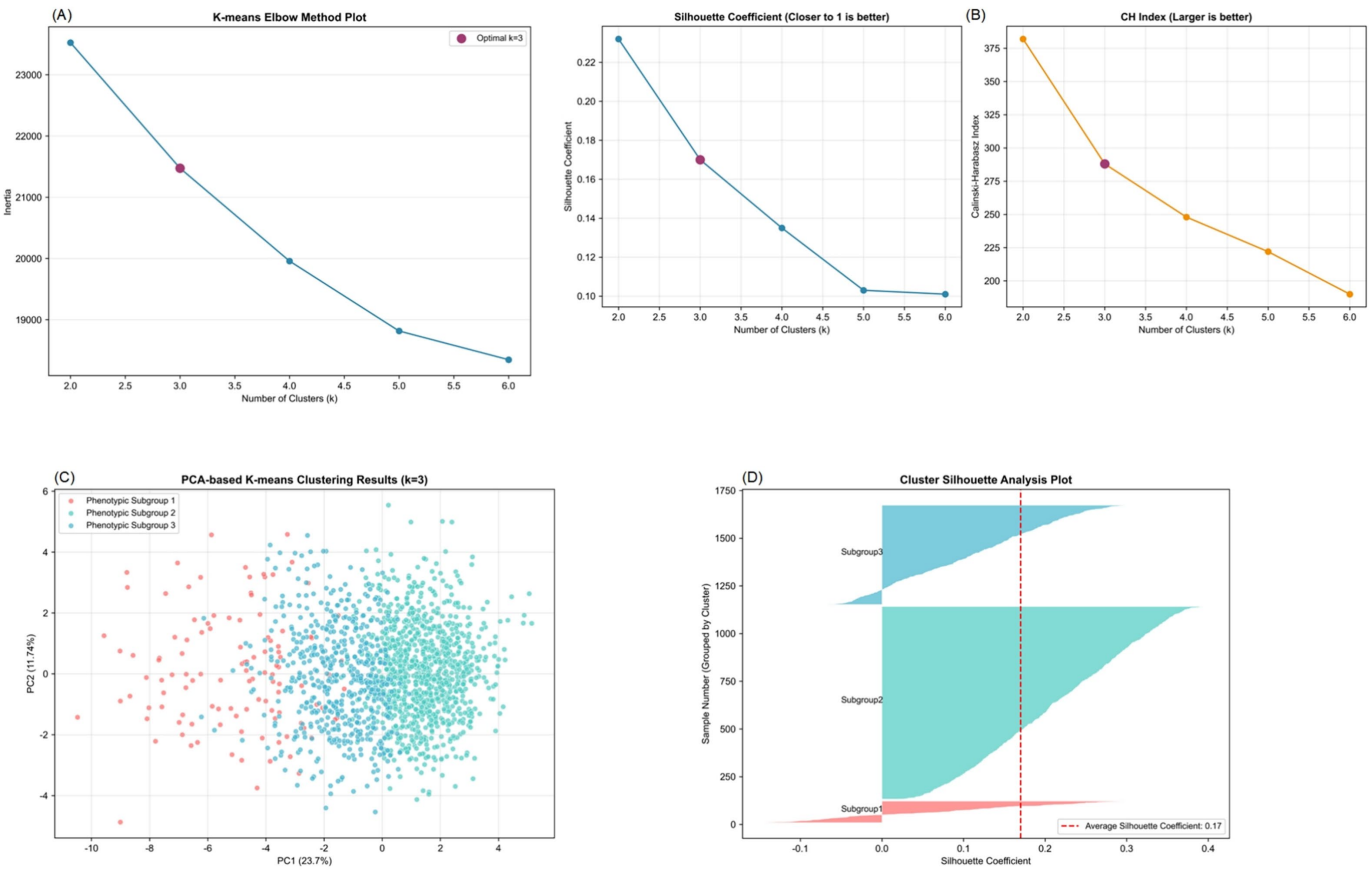
Determination of the optimal number of K-means clusters and validation of the clustering structure. **(A)** Elbow plot showing within-cluster inertia values across different cluster numbers. The purple marker indicates the optimal cluster number (k = 3); **(B)** Silhouette coefficient and Calinski–Harabasz (CH) index values plotted against the cluster number k. The purple marker indicates the selected optimal cluster number (k = 3); **(C)** Patient subgroup distribution in the PC1–PC2 score plot. Subgroup 1 (red), Subgroup 2 (green), and Subgroup 3 (cyan) are shown in distinct colors; **(D)** Silhouette analysis for the k = 3 clustering solution. Each bar represents the silhouette coefficient of one patient; the red dashed line indicates the mean silhouette width (0.17). Bar colors correspond to subgroup assignments shown in panel C.

The optimal cluster number was determined to be k = 3 based on three complementary metrics. The elbow method identified a clear inflection point at k = 3, with the within-cluster sum of squares declining from approximately 23,500 at k = 2 to 21,400 at k = 3, followed by a plateau at k ≥ 4. The CH index at k = 3 (288.0) was higher than at k ≥ 4. Notably, the silhouette coefficient at k = 2 (0.232) was numerically higher than at k = 3 (0.170); however, the silhouette coefficient has an inherent tendency to favor coarser partitions (23). In clinical datasets where biological variables exist on a continuum, moderate silhouette values are expected and do not invalidate the clustering structure (24). The relevant clustering diagnostic indices are presented in Supplementary Table 3, and the results of cluster number determination and validation are shown in Figures 3A,B.

#### 3.5.2 Clustering results and validation

Based on the clustering solution with k = 3, patients were stratified into three distinct subgroups (Figure 3C), which exhibited clear spatial separation in the PC1-PC2 score plot. Subgroup 1 (red) was predominantly concentrated in the negative region of the PC1 axis, whereas Subgroup 2 (green) was mainly located in the positive region. Subgroup 3 (cyan) was densely clustered around the origin. This distribution pattern indicated that PC1 was the primary dimension driving subgroup differentiation, with the three clusters displaying a continuous gradient from low to high along the PC1 axis—reflecting core host characteristics such as inflammatory and nutritional status.

Silhouette analysis confirmed the validity of the clustering structure, yielding an overall average silhouette coefficient of 0.17 (Figure 3D). Subgroups 2 and 3 showed predominantly positive silhouette coefficients, indicating high intra-cluster homogeneity and good separation. Although most samples in Subgroup 1 had positive coefficients, a few values were close to zero, suggesting slight heterogeneity within this cluster.

### 3.6 Identification and characteristics of phenotypic subgroups

#### 3.6.1 Heterogeneity in clinical characteristics

Unsupervised clustering analysis divided the 1,644 patients with advanced NSCLC into three phenotype subgroups (Tables 4, 5). Significant heterogeneity was observed across multiple key clinical variables among the subgroups (*p* < 0.05). Subgroup 1 (*n* = 113, 6.87%) had the highest cachexia rate (41.6%), the highest proportion of male patients (65.5%), and the highest smoking rate (59.3%). Subgroup 2 (*n* = 1,010, 61.44%) had the highest proportion of squamous cell carcinoma (70.9%) and the youngest mean age (65.4 ± 9.7 years). Subgroup 3 (*n* = 521, 31.69%) had a mean age similar to Subgroup 1 (69.2 ± 9.7 years) and a higher alcohol consumption rate (40.7%) than Subgroup 2 (31.2%; *p* < 0.001). Tumor stage did not differ significantly among the three subgroups (χ^2^ = 2.878, *p* = 0.578), indicating that this phenotypic classification was independent of TNM staging (Table 5). Regarding missing sex data, there was no significant difference in the rate of missing sex data among the three phenotypic subgroups (6.2, 11.2, and 12.3%; χ^2^ = 3.63, *p* = 0.163), suggesting that missing sex status was not significantly associated with subgroup membership. Further sensitivity analysis among patients with known sex (*n* = 1,460) showed that differences in the proportion of male patients among the subgroups remained significant (69.8, 57.2, and 63.0%; χ^2^ = 8.72, *p* = 0.013), consistent with the direction of the primary analysis (Supplementary Table 4).

**Table 4:** Distribution of patients across phenotypic subgroups in advanced NSCLC.

Phenotypic subgroup	Sample number (n)	Proportion (%)
Subgroup 1	113	6.87
Subgroup 2	1,010	61.44
Subgroup 3	521	31.69
Total	1,644	100.00

A total of 1,644 patients with advanced NSCLC were classified into three phenotypic subgroups using unsupervised clustering (PCA combined with K-means).

**Table 5:** Comparison of clinical characteristics among different phenotypic subgroups in advanced NSCLC.

Variable	Subgroup 1 (*n* = 113)	Subgroup 2 (*n* = 1,010)	Subgroup 3 (*n* = 521)	*F*/χ^2^	*p*-value
Age (years), mean ± SD	69.0 ± 10.0	65.4 ± 9.7	69.2 ± 9.7	30.234	< 0.001***
Gender, *n* (%)	**–**	**–**	**–**	12.614	0.013*
Male	74 (65.5)	513 (50.8)	288 (55.3)	**–**	**–**
Female	32 (28.3)	384 (38.0)	169 (32.4)	**–**	**–**
Unknown	7 (6.2)	113 (11.2)	64 (12.3)	**–**	**–**
Pathological type, *n* (%)	**–**	**–**	**–**	56.434	< 0.001***
Squamous cell carcinoma	54 (47.8)	716 (70.9)	288 (55.3)	**–**	**–**
Adenocarcinoma	33 (29.2)	190 (18.8)	129 (24.8)	**–**	**–**
Other	26 (23.0)	104 (10.3)	104 (20.0)	**–**	**–**
Tumor stage, *n* (%)	**–**	**–**	**–**	2.878	0.578
II	1 (0.9)	14 (1.4)	7 (1.3)	**–**	**–**
III	46 (40.7)	411 (40.7)	190 (36.5)	**–**	**–**
IV	66 (58.4)	585 (57.9)	324 (62.2)	**–**	**–**
Smoking history, *n* (%)	**–**	**–**	**–**	29.372	< 0.001***
Yes	67 (59.3)	427 (42.3)	287 (55.1)	**–**	**–**
No	46 (40.7)	583 (57.7)	234 (44.9)	**–**	**–**
Drinking history, *n* (%)	**–**	**–**	**–**	15.034	< 0.001***
Yes	45 (39.8)	315 (31.2)	212 (40.7)	**–**	**–**
No	68 (60.2)	695 (68.8)	309 (59.3)	**–**	**–**
Cachexia, *n* (%)	**–**	**–**	**–**	65.058	< 0.001***
Yes	47 (41.6)	186 (18.4)	180 (34.5)	**–**	**–**
No	66 (58.4)	824 (81.6)	341 (65.5)	**–**	**–**
NRS-2002 score, median (IQR)	3 (2–4)	2 (2–3)	3 (2–3)	**–**	<0.001
NRS-2002 ≥ 3, *n* (%)	73 (64.6)	351 (34.8)	291 (55.9)	χ^2^ = 84.28	<0.001

1. Continuous variables were analyzed using one-way ANOVA, and categorical variables were analyzed using the chi-square test. 2. *p < 0.05; ***p < 0.001. 3. “Other” types included NSCLC-not otherwise specified (NSCLC-NOS) and other rare histological subtypes of non-small cell lung cancer not classified as adenocarcinoma or squamous cell carcinoma. Due to the limited number of cases, these patients were not analyzed as an independent subgroup.

#### 3.6.2 Molecular phenotypic characteristics based on laboratory indicators

**Figure 4:**
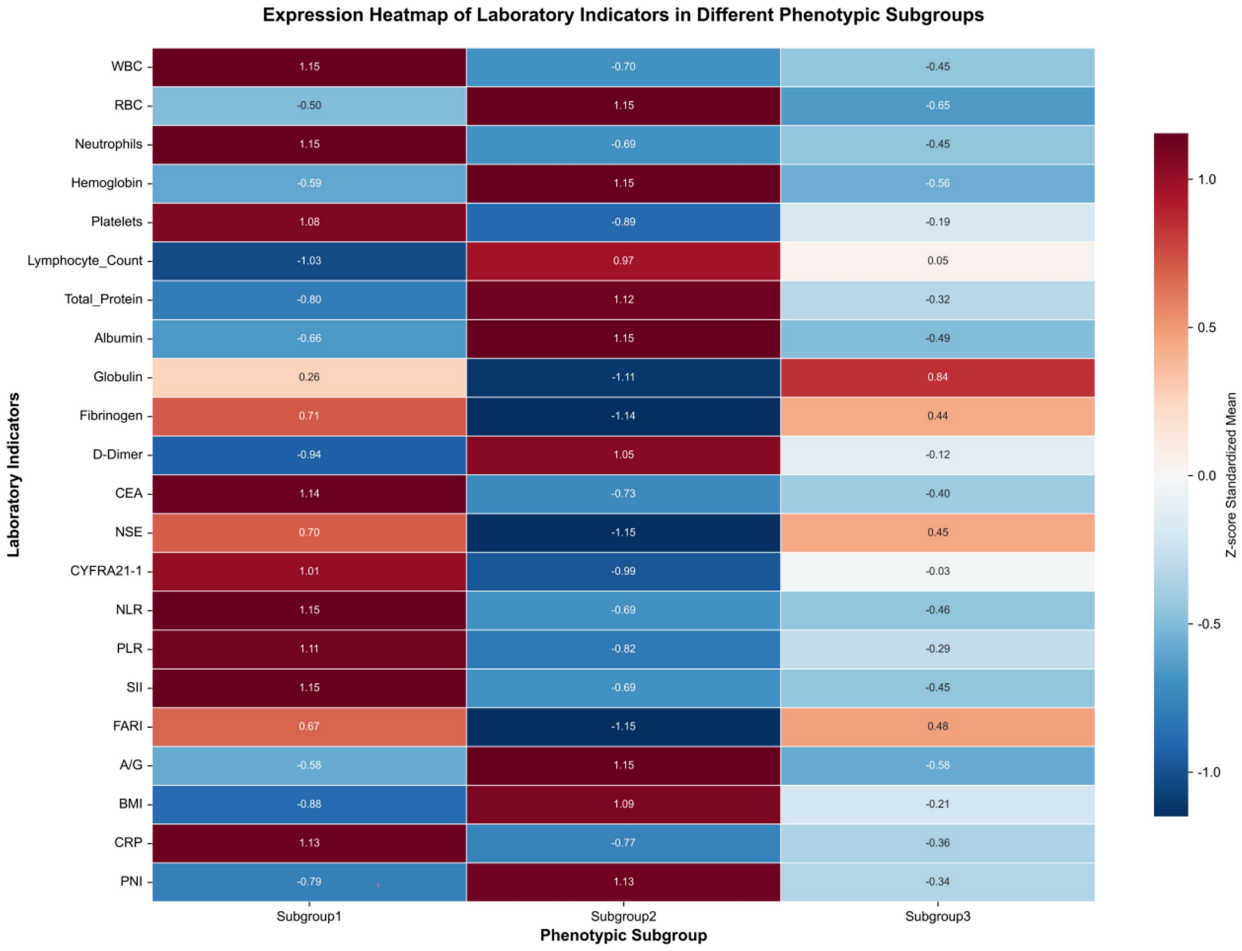
Heatmap of laboratory indicators across different phenotypic subgroups. Each row represents a laboratory indicator and each column represents a phenotypic subgroup. Color indicates Z-score: red represents high expression (Z > 1.0) and blue represents low expression (Z < −0.8). FARI, fibrinogen-to-albumin ratio index; A/G, albumin-to-globulin ratio; WBC, white blood cell count; RBC, red blood cell count; CRP, C-reactive protein. Subgroup 1: hyper-inflammatory, hypo-nutritional, and hyper-coagulable phenotype; Subgroup 2: intermediate phenotype approximating the cohort mean; Subgroup 3: hypo-inflammatory, hyper-nutritional, and hypo-coagulable phenotype.

**Figure 5:**
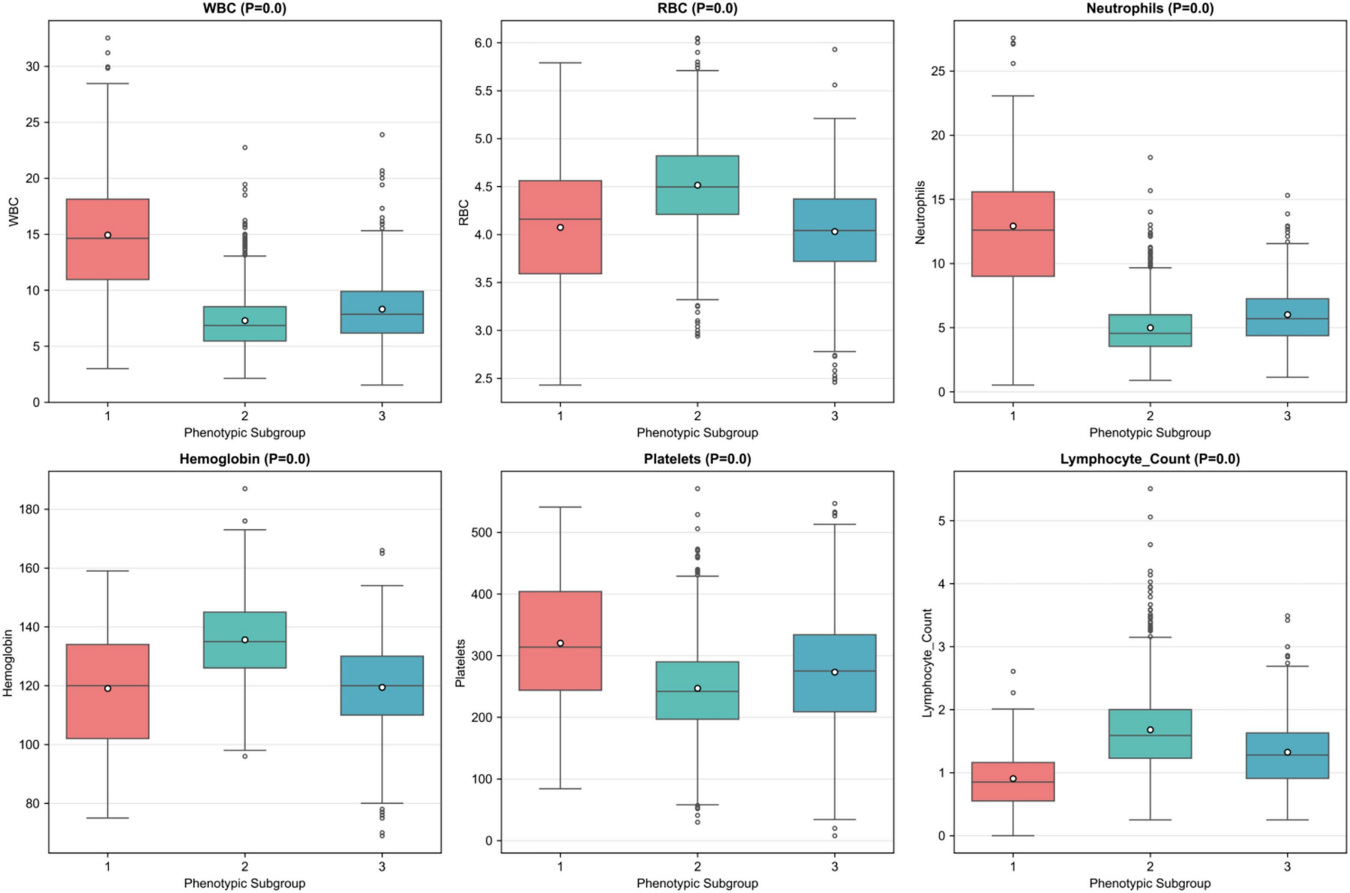
Boxplots of key hematological parameters across phenotypic subgroups. Boxplots in red, turquoise, and cyan represent subgroups 1, 2, and 3, respectively. The white dot within each box indicates the median. The box bounds represent the interquartile range (IQR, Q1–Q3), and the whiskers extend to 1.5 × the IQR. Individual points beyond the whiskers represent outliers. Statistically significant differences were observed among the three subgroups for all displayed indices (*p* < 0.05). WBC, white blood cell count; RBC, red blood cell count.

**Figure 6:**
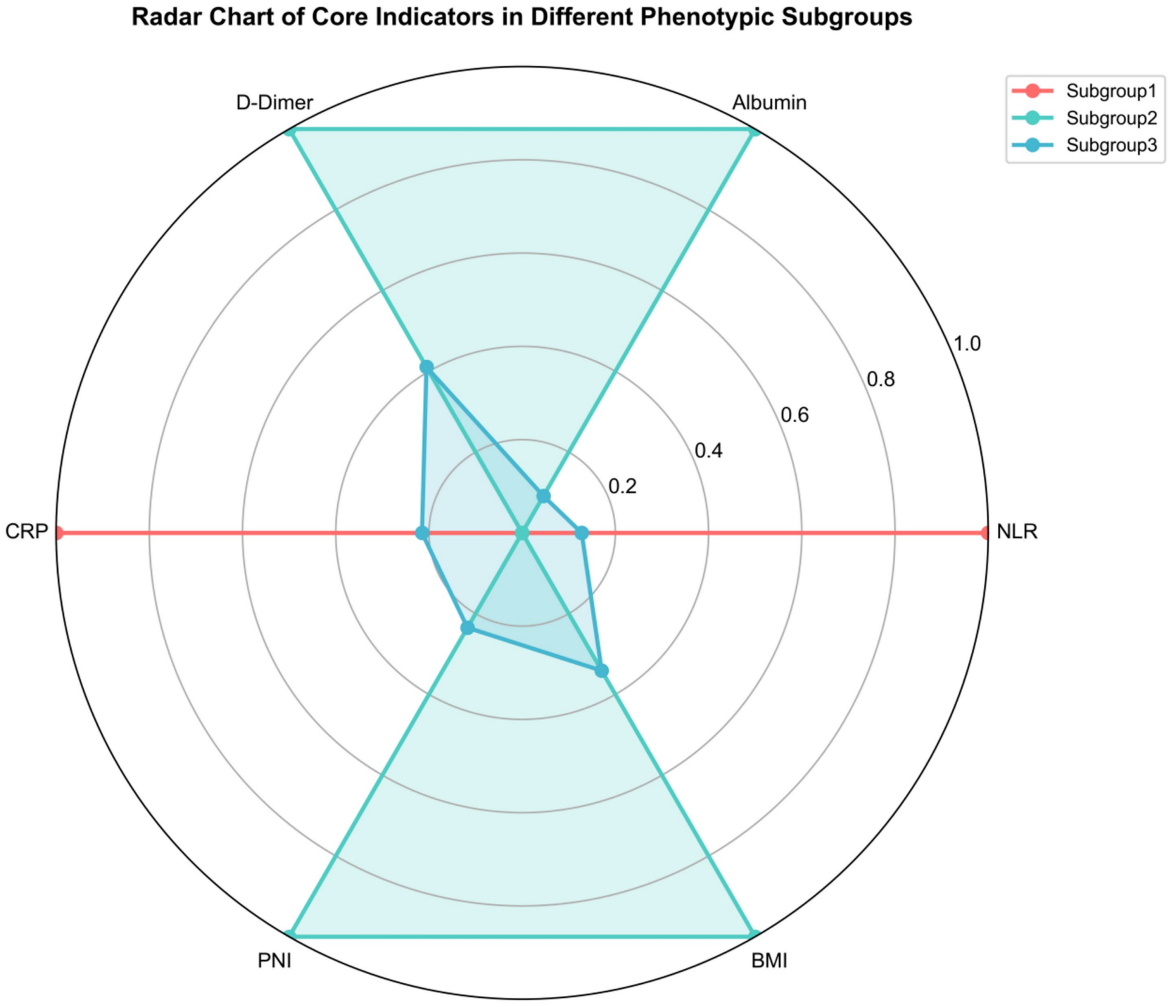
Radar chart of inflammation–nutrition–coagulation phenotypic profiles across subgroups in advanced NSCLC. Z-score standardized values of six core indicators (NLR, albumin, BMI, PNI, CRP, and D-dimer). Different colors represent the three subgroups. Radial distance reflects the relative level of each indicator. CRP, C-reactive protein.

Analysis of laboratory indicators revealed that the three subgroups exhibited distinct integrated phenotypic profiles encompassing inflammatory, nutritional, and coagulation axes (Figures 4–6). Significant intergroup differences were observed across all core laboratory parameters (*p* < 0.05).

The heatmap (Figure 4) illustrates that Subgroup 1 was characterized by a hyper-inflammatory, hypo-nutritional, and hyper-coagulable phenotype. This profile was defined by the marked elevation of inflammatory markers (neutrophils, NLR, SII, CRP), coagulation indices (fibrinogen, D-dimer), and tumor markers (CEA, CYFRA 21-1; Z-score > 1.0), concomitant with a significant reduction in nutritional and immune parameters (lymphocyte count, albumin and PNI; Z-score < −0.8). Conversely, Subgroup 3 displayed a hypo-inflammatory, hyper-nutritional, and hypo-coagulable phenotype, presenting a pattern inverse to that of Subgroup 1, with normalized inflammatory and coagulation indices and robust nutritional and immune parameters. Subgroup 2 represented an intermediate phenotype, with indicator levels approximating the cohort mean (Z-score ≈ 0) and no overt abnormalities in inflammation, nutrition, or coagulation function.

Quantitative comparisons of hematological parameters (Figure 5) further corroborated these phenotypic distinctions. Subgroup 1 exhibited the highest median WBC and neutrophil count, alongside the lowest median lymphocyte count, red blood cell count, and hemoglobin levels. Subgroup 3 demonstrated opposite trends, while Subgroup 2 maintained intermediate values (all *p* < 0.05).

A radar chart visualization (Figure 6), based on six core indicators (NLR, albumin, BMI, PNI, CRP and D-dimer), provided comprehensive integration of these phenotypic profiles. Subgroup 1 showed a distinct skew toward inflammatory and coagulation axes with a concurrent depression in nutritional axes; Subgroup 3 exhibited a reciprocal profile; and Subgroup 2 clustered near the central region, reflecting a balanced physiological state.

#### 3.6.3 Comparison of hospitalization-related outcomes

Hospitalization-related outcomes differed significantly among the phenotypic subgroups (Table 6). Patients in Subgroup 1 had the highest hospitalization burden, whereas those in Subgroup 3 had the lowest; the between-group differences in length of hospital stay and the incidence of prolonged hospitalization were both statistically significant (both *p* < 0.001).

**Table 6:** Comparison of hospitalization-related outcomes among different phenotypic subgroups.

Variable subgroup	Subgroup 1 (*n* = 113)	Subgroup 2 (*n* = 1,010)	Subgroup 3 (*n* = 521)	*F*/χ^2^	*p*-value
Length of hospital stay (d, x̄±s)	10.9 ± 3.4	9.6 ± 3.1	8.8 ± 3.3	*F* = 23.51	<0.001
Prolonged hospitalization, *n* (%)	55 (48.7)	374 (37.0)	157 (30.1)	χ^2^ = 16.01	<0.001

Continuous variables were analyzed using one-way analysis of variance; categorical variables were analyzed using the χ^2^ test. Prolonged hospitalization was defined as a length of hospital stay exceeding 10 days.

## 4 Discussion

The pronounced prognostic heterogeneity among patients with advanced NSCLC remains a central challenge in precision oncology (25). While the traditional TNM staging system provides a fundamental framework for treatment decision-making based on anatomical tumor characteristics, it fails to account for the substantial variability in therapeutic response and survival outcomes observed among patients with the same stage (26). This limitation highlights the critical need for precise patient stratification based on the host’s systemic biological status. The dynamically interactive network comprising inflammation, nutrition, and coagulation systems has been recognized as a key host factor regulating tumor progression, metastasis, and therapeutic resistance (14). Consequently, host phenotype analysis integrating multi-system indicators offers a novel perspective for deciphering the clinical heterogeneity of advanced NSCLC.

This study found that the phenotypic classification was not associated with TNM staging (*p* = 0.578), suggesting that host systemic status may represent an independent dimension of clinical heterogeneity in advanced NSCLC. Subgroup 1 (6.87%) showed an extreme phenotype of high inflammation, poor nutrition, and hypercoagulability, providing a new biological basis and potential intervention targets for risk stratification and individualized treatment. This study combined PCA dimensionality reduction with K-means clustering, overcoming limitations of single-marker methods and conventional regression models (27, 28). The nine extracted principal components effectively captured the complex interactions and multicollinearity among the three systems. The moderate silhouette coefficient (0.17) reflects a continuous gradient of systemic biological status in advanced NSCLC rather than discrete categorical boundaries (24). As Rousseeuw showed, moderate values do not invalidate clustering structure in clinical datasets where biological variables exist on a continuum (23). The agreement among three validation metrics supported the robustness of the k = 3 solution. Significant inter-subgroup differences in cachexia rate, NLR, and albumin (all *p* < 0.05) further confirmed its clinical interpretability. This approach relies only on routine laboratory indicators, offering a practical and reproducible method for examining biological heterogeneity in tumor hosts.

The three phenotypic subgroups identified represent distinct systemic biological states in advanced NSCLC. Integrating evidence from existing multi-omics studies allows us to preliminarily elucidate their molecular biological basis, providing theoretical support for personalized intervention strategies. Subgroup 1, identified as a high-risk phenotype, essentially reflects a systemic pathophysiological vicious cycle driven by tumor-induced multi-system disorders (29). This subgroup exhibited significantly elevated inflammatory markers, markedly reduced nutritional and immune indicators, and abnormal coagulation function, consistent with the characteristics of systemic inflammation-centered protein-energy wasting syndrome in tumor-related malnutrition. Regarding the immune microenvironment, transcriptomic studies confirm that tumors from patients with high NLR and CRP phenotypes are enriched in immunosuppressive cells, such as M2-type macrophages and regulatory T cells (Treg), with high expression of immune checkpoint molecules (PD-L1, CTLA-4) and inflammatory factors (IL-6, TNF-*α*), accompanied by exhausted effector T cells (30, 31). This aligns closely with the inflammatory activation profile of Subgroup 1. In terms of metabolic reprogramming, metabolomic data demonstrate that patients with tumor-related malnutrition exhibit an enhanced Warburg effect and upregulated glutamine metabolism. This leads to the competitive consumption of host nutritional substrates and hyperactive protein catabolism (32, 33), adequately explaining the significantly reduced albumin, PNI, and lymphocyte counts observed in this subgroup. At the molecular pathway level, proteomic studies indicate that hypercoagulability is closely associated with activation of the PI3K/Akt/mTOR pathway, which promotes tissue factor (TF) expression and procoagulant release. Concurrently, inflammation-mediated NF-κB pathway activation further exacerbates vascular endothelial injury (34), consistent with the elevated D-dimer and fibrinogen levels in Subgroup 1. Conventional nutritional support may be insufficient to suppress inflammation-driven catabolism in this phenotype. A dual-target strategy combining anti-inflammatory and pro-anabolic nutritional interventions may therefore be warranted (35). Based on adequate protein intake, the combined use of immunonutritional formulas rich in *ω*-3 fatty acids may regulate inflammatory responses and inhibit protein breakdown (36). Meanwhile, the immunosuppressive profile suggests a potentially poor response to immune checkpoint inhibitor therapy, warranting careful clinical evaluation of therapeutic benefits (37).

Subgroup 3 represented a favorable phenotype, characterized by normal inflammatory markers, significantly elevated nutritional and immune indicators, and no activation of coagulation function, indicating a state dominated by anabolic metabolism. At the immune microenvironment level, proteomic studies show that tumors from patients with low-inflammatory and high-nutritional phenotypes exhibit abundant infiltration of CD8^+^ T cells and natural killer (NK) cells, low expression of immune checkpoint molecules, and preserved anti-tumor immune surveillance (38). This is consistent with the relatively high lymphocyte count in Subgroup 3. Regarding metabolic characteristics, genomic studies confirm that patients with driver gene mutations such as ALK fusion or ROS1 rearrangement maintain intact mitochondrial oxidative phosphorylation without overt tumor-induced metabolic reprogramming disorders (39). Consequently, their nutritional indicators (albumin, PNI) usually remain at high levels, mirroring the phenotype of Subgroup 3. At the pathway level, inflammatory-mediated signaling pathways are only mildly activated, and coagulation-related pathways are not abnormally upregulated, precluding a hypercoagulable state (40). The favorable nutritional status provides sufficient metabolic support for T-cell activation and proliferation, while normal coagulation reduces the physical barrier of microthrombosis to effector T-cell infiltration (41), suggesting this subgroup is a potentially favorable population for immune checkpoint inhibitor therapy. Nutritional management for this subgroup focuses on maintaining this favorable status through a balanced diet and appropriate protein intake to prevent iatrogenic malnutrition caused by chemotherapy and radiotherapy.

Subgroup 2 exhibited an intermediate phenotype, with all laboratory parameters approximating the overall cohort mean and no overt abnormalities in inflammation, nutrition, or coagulation function. This represents a transitional state between Subgroup 1 and Subgroup 3. At the transcriptomic level, existing studies indicate that patients with intermediate phenotypes exhibit expression levels of inflammatory factors (IL-6, IL-8) and immune checkpoint molecules that are intermediate between high-risk and favorable phenotypes. No significant polarization of immune cell function has been observed (42). Metabolically, glycolysis and oxidative phosphorylation pathways are in dynamic equilibrium without obvious metabolic disturbance (43). Genomically, such patients mostly harbor wild-type tumors or low-frequency driver gene mutations, lacking dominant malignant biological characteristics (44), which is consistent with the phenotype of Subgroup 2. This subgroup exists in a compensatory state, presenting only mild inflammatory activation and nutritional consumption without falling into a systemic vicious cycle, thus representing a critical window for clinical intervention. Timely screening using the NRS2002 nutritional risk screening tool and the initiation of interventions such as oral nutritional supplements are expected to prevent progression to the high-risk phenotype (Subgroup 1). This approach may improve tolerance and response to subsequent anti-tumor therapy (45).

This phenotypic classification system has considerable translational potential. Existing inflammation-based scoring tools such as the Glasgow Prognostic Score and modified GPS, which use CRP and albumin to stratify cancer patients, have shown independent prognostic value in advanced NSCLC (46). This finding suggests that building clinical scoring tools from host status indicators is feasible. The present study extends this approach by incorporating a broader set of biomarkers across the inflammation–nutrition–coagulation axis, providing data to support the development of more refined tools; however, their prognostic value requires prospective validation. In practice, a simplified bedside scoring tool could be built from core indicators such as NLR, albumin, CRP, and D-dimer, using cluster centroids as threshold references for rapid identification of high-risk patients. Combining host phenotype with TNM staging may support a more precise two-dimensional prognostic model. Most importantly, this classification offers a hypothetical framework for precision nutritional management, pending prospective validation: Subgroup 1 may benefit from aggressive nutritional and anti-inflammatory intervention, Subgroup 3 may benefit from maintenance nutritional support to prevent iatrogenic deterioration, and Subgroup 2 may represent a potential window for early nutritional screening and preventive supplementation. In terms of clinical accessibility, most variables included in this study, including complete blood count parameters, biochemical indices, and coagulation-related markers, are routinely assessed during the inpatient evaluation of patients with lung cancer. Only a limited number of tumor markers, such as CEA, NSE, and CYFRA 21-1, may not be routinely available in some primary care or resource-limited settings. Therefore, the additional testing burden required for practical implementation of the model is relatively limited. Meanwhile, the proposed stratification framework should be regarded as an exploratory tool. Future studies will focus on identifying a reduced subset of representative core variables and developing a simplified and more generalizable clinical scoring system through multicenter prospective validation, thereby improving the model’s applicability and clinical utility.

From the perspective of clinical nutrition, the three host phenotypes identified in this study exhibited distinct nutritional risk stratification characteristics. The NRS-2002 results showed that 64.6% of patients in Subgroup 1 were at nutritional risk (NRS-2002 ≥ 3), which was significantly higher than that in Subgroup 2 (34.8%), suggesting a high degree of concordance between this subgroup and the high nutritional risk population. This finding was consistent with the inflammatory activation, reduced albumin levels, decreased PNI, and high prevalence of cachexia observed in this subgroup. Previous studies have shown that cancer-related malnutrition is closely associated with reduced treatment tolerance, increased complications, decreased quality of life, and poor prognosis (47, 48). Therefore, patients in Subgroup 1 should be considered a priority population for nutritional intervention. Systematic nutritional assessment should be initiated as early as possible, and individualized nutritional support strategies should be implemented in accordance with the ESPEN guidelines, including nutritional counseling, oral nutritional supplements, and intensive nutritional support when necessary (49).

In contrast, the proportion of patients at nutritional risk was lower in Subgroup 2, suggesting that its overall nutritional status was relatively stable; however, routine screening and dynamic monitoring are still required during antitumor treatment to prevent progression to a high-risk phenotype. Although Subgroup 3 exhibited more favorable inflammatory and nutritional indicator profiles, 55.9% of patients still met the NRS-2002 criteria for nutritional risk, indicating that clinical nutritional risk cannot be excluded even when laboratory nutritional indicators are relatively favorable. Therefore, the primary goals for this subgroup should be to maintain nutritional status and prevent treatment-related malnutrition.

Notably, the phenotypic classification proposed in this study is not intended to replace existing nutritional screening tools (such as NRS-2002, PG-SGA, and MUST), but rather to complement conventional nutritional assessment systems (50–52). By integrating inflammation-, nutrition-, and coagulation-related indicators, this classification framework may provide more refined nutritional risk stratification for patients with advanced NSCLC, thereby optimizing individualized nutritional management pathways.

The lack of a significant association between phenotypic subgroups and TNM stage (*p* = 0.578) is noteworthy but requires careful interpretation. While this finding suggests that host systemic status constitutes an independent dimension of clinical heterogeneity, several points warrant caution. First, this was a cross-sectional baseline analysis and does not establish independent prognostic value after adjustment for TNM stage. Second, the absence of survival data means it is not possible to determine whether these phenotypes carry additional prognostic value. Third, the high proportion of stage III–IV patients (98.7%) limits the TNM range covered, which may reduce the sensitivity of this comparison. Prospective studies with survival endpoints are needed to confirm the independent prognostic contribution of host phenotype in a multivariable framework.

Although this study lacked survival outcomes, significant differences in hospitalization-related outcomes were observed among the different phenotypic subgroups. Subgroup 1 had a greater hospitalization burden, whereas Subgroup 3 had relatively more favorable hospitalization outcomes. This finding was consistent with the high inflammatory state, malnutrition, and hypercoagulability observed in Subgroup 1. Although hospitalization-related indicators cannot replace long-term prognostic endpoints, they suggest that the identified host phenotypes may have clinically meaningful consequences beyond differences in laboratory indicators, thereby providing preliminary evidence for the potential application of phenotypic stratification in the management of patients with advanced NSCLC.

This study has several limitations. First, the single-center retrospective design carries an inherent risk of selection bias and limits causal inference; multi-center prospective studies are needed to confirm the generalizability of the findings. The exclusion of patients with hematological disorders, severe cardiovascular disease, and conditions that may confound inflammatory or coagulation parameters may have led to systematic underrepresentation of patients with extreme phenotypic profiles, potentially resulting in underestimation of the prevalence and severity of the high-risk phenotype (Subgroup 1). In this study, the proportion of squamous cell carcinoma (64.4%) was higher than that reported in global epidemiological data, where lung adenocarcinoma is the predominant histological subtype. This discrepancy may be attributed to the single-center retrospective design in northern China, with a study population predominantly composed of elderly male patients with a high prevalence of smoking history, among whom smoking-related lung cancer is more frequently associated with squamous cell carcinoma. In addition, squamous cell carcinoma is more commonly centrally located and is therefore more readily diagnosed via bronchoscopic biopsy, which may lead to its relative enrichment in hospital-based and pathology-derived cohorts. These factors suggest that the findings should be interpreted with caution when extrapolated to populations with a higher prevalence of lung adenocarcinoma or different geographic settings. Second, as this study was based on cross-sectional baseline data, the observed phenotypic differences reflect associations rather than causal relationships; whether modifying host phenotype translates into improved outcomes requires prospective interventional studies. Third, potential confounding factors were not systematically controlled and may have influenced the observed phenotypic differences. These include specific comorbidities such as diabetes and chronic obstructive pulmonary disease, concurrent medications such as anticoagulants, corticosteroids, and non-steroidal anti-inflammatory drugs, as well as performance status. Fourth, survival endpoints were not included; the independent prognostic value of each subgroup requires confirmation in prospective studies with survival data, alongside development of a combined prognostic model integrating host phenotype and TNM staging. Fifth, only static baseline indicators were analyzed; longitudinal studies are needed to examine phenotype evolution during treatment and to assess the feasibility of using phenotype as a dynamic monitoring tool. Sixth, mechanistic exploration relied on published literature and correlation analysis rather than direct experimental evidence; future work should integrate multi-omics approaches to clarify the underlying mechanisms (53).

## 5 Conclusion

Using PCA combined with K-means clustering, this study identified three distinct host phenotype subgroups in advanced NSCLC based on inflammatory, nutritional, and coagulation biomarkers. These subgroups were independent of TNM staging and provide a new biological framework for risk stratification. Multi-center validation, survival analysis, and mechanistic investigation will be essential to advance this approach toward clinical use.

## Data Availability

The raw data supporting the conclusions of this article will be made available by the authors, without undue reservation.
