# Supplementary material for: Clinical phenotype identification based on inflammation–nutrition–coagulation biomarkers in advanced non-small cell lung cancer
Source: Front Nutr. 2026 Jul 15;13:1839434. doi: 10.3389/fnut.2026.1839434 (PMC13414893; doi:10.3389/fnut.2026.1839434)
Supplement: Supplementary file 1 [file Table_1.DOCX]

Supplementary Material

**Supplementary Table 1. Results of PCA adequacy testing**

| Test | Statistic | Value | P-value |
| --- | --- | --- | --- |
| KMO test | **–** | 0.631 | **–** |
| Bartlett's test of sphericity | χ^2^ | 30965.06 | P<0.001 |

**Supplementary Table 2. Eigenvalues and explained variance for the extracted principal components**

| Principal component | Eigenvalue | Variance contribution rate (%) | Cumulative variance contribution rate (%) |
| --- | --- | --- | --- |
| PC1 | 5.217 | 23.70 | 23.70 |
| PC2 | 2.585 | 11.74 | 35.44 |
| PC3 | 2.247 | 10.21 | 45.65 |
| PC4 | 1.666 | 7.57 | 53.22 |
| PC5 | 1.539 | 6.99 | 60.21 |
| PC6 | 1.236 | 5.62 | 65.82 |
| PC7 | 1.109 | 5.04 | 70.86 |
| PC8 | 1.041 | 4.73 | 75.59 |
| PC9 | 1.011 | 4.59 | 80.18 |
| PC10 | 0.958 | 4.35 | 84.54 |

Note: Principal components were extracted based on the Kaiser criterion (eigenvalues > 1), with a cumulative explained variance of 80.18%

**Supplementary Table 3. K-means clustering validity metrics for different cluster numbers**

| Number of clusters k | Silhouette coefficient | CH index |
| --- | --- | --- |
| 2 | 0.232 | 382.0 |
| 3 | 0.170 | 288.0 |
| 4 | 0.135 | 248.0 |
| 5 | 0.103 | 222.0 |
| 6 | 0.101 | 190.0 |

**Supplementary Table 4. Missingness of sex variable and sensitivity analysis restricted to patients with known sex**

| Analysis item | Subgroup 1 | Subgroup 2 | Subgroup 3 | *χ²* | *P* value |
| --- | --- | --- | --- | --- | --- |
| Missing sex information, n (%) | 7 (6.2) | 113 (11.2) | 64 (12.3) | 3.63 | 0.163 |
| Male proportion among patients with known sex, n/N (%) | 74/106 (69.8) | 513/897 (57.2) | 288/457 (63.0) | 8.72 | 0.013 |
